# Glacial lakes exacerbate Himalayan glacier mass loss

**DOI:** 10.1038/s41598-019-53733-x

**Published:** 2019-12-02

**Authors:** Owen King, Atanu Bhattacharya, Rakesh Bhambri, Tobias Bolch

**Affiliations:** 1 Department of Geography, University of Zurich, Zurich, CH-8057 Switzerland; 2 School of Geography & Sustainable Development, University of St. Andrews, Scotland, KY16 9AL UK; 3 Centre for Glaciology, Wadia Institute of Himalayan Geology, Dehradun, India 248001

**Keywords:** Climate change, Cryospheric science

## Abstract

Heterogeneous glacier mass loss has occurred across High Mountain Asia on a multi-decadal timescale. Contrasting climatic settings influence glacier behaviour at the regional scale, but high intra-regional variability in mass loss rates points to factors capable of amplifying glacier recession in addition to climatic change along the Himalaya. Here we examine the influence of surface debris cover and glacial lakes on glacier mass loss across the Himalaya since the 1970s. We find no substantial difference in the mass loss of debris-covered and clean-ice glaciers over our study period, but substantially more negative (−0.13 to −0.29 m w.e.a^−1^) mass balances for lake-terminating glaciers, in comparison to land-terminating glaciers, with the largest differences occurring after 2000. Despite representing a minor portion of the total glacier population (~10%), the recession of lake-terminating glaciers accounted for up to 32% of mass loss in different sub-regions. The continued expansion of established glacial lakes, and the preconditioning of land-terminating glaciers for new lake development increases the likelihood of enhanced ice mass loss from the region in coming decades; a scenario not currently considered in regional ice mass loss projections.

## Introduction

Glacier mass loss has occurred across large parts of High Mountain Asia over at least the last four decades^1–4^, although substantial spatial variability has been documented in the magnitude of glacier mass loss in the region. Glaciers in the Karakoram, Kunlun Shan and eastern Pamir have maintained mass balance to the present day^3,5–7^, whereas glaciers located in the Himalaya, in the Tien Shan and Nyainqentanghla have experienced substantial mass loss in recent decades^2,6^. The disparity in regional mass loss rates has been attributed to the diminished sensitivity to warming of glaciers in the Karakoram, Kunlun Shan and eastern Pamir due to their accumulation of snowfall in winter months, rather than during the summer monsoon along the Himalaya^8^. However, large intra-regional variability in glacier mass loss is evident along the Himalayan arc^6,9^, which suggests factors exist that are capable of exacerbating glacier recession in addition to climatic change here.

Glaciers situated in the Himalaya commonly have extensive debris cover^10^, and an increasing number terminate into a glacial lake^11^. A continuous debris mantle thicker than a few centimetres dampens sub-debris ablation rates^12^. Modelling studies have shown how debris cover enables the persistence of greater glacier area in comparison with clean-ice in a changing climate^13,14^. However, comparable thinning rates have been observed for clean-ice and debris-covered glaciers at similar elevations^15–17^ at several locations in the Himalaya.

Glacial lakes amplify ice loss from their host glaciers through mechanical calving and subaqueous melt^18,19^. There are currently more than 700 proglacial lakes in the Himalaya^11,20^, which are all capable of directly influencing the behaviour of their host glacier. Proglacial lake area expanded by >50% in the Himalaya between 1990 and 2015^11^. Enhanced glacier area reductions have been observed from lake-terminating glaciers in the Sikkim Himalaya^21^, and elevated glacier mass loss from lake-terminating glaciers has recently been confirmed as a region-wide phenomenon^9,22,23^. However, a comprehensive analysis of the impact of glacial lakes on glacier retreat and mass loss rates in the Himalaya is still lacking.

The main aim of this study is therefore to examine the influence of a debris mantle and glacial lake development on the long-term evolution of Himalayan glaciers in detail, in order to improve our understanding of the regional variability of ice loss rates. We quantify mass loss and terminus retreat from lake and land-terminating glaciers along the Himalayan arc since the 1970s, using optical and radar based remotely-sensed datasets. We use these data to discuss the role of debris cover and glacial lakes as drivers of glacier mass loss in the Himalaya and consider the future evolution of glaciers in the region.

## Glacier Mass Balance and Ice Front Retreat Rates

**Figure 1:**
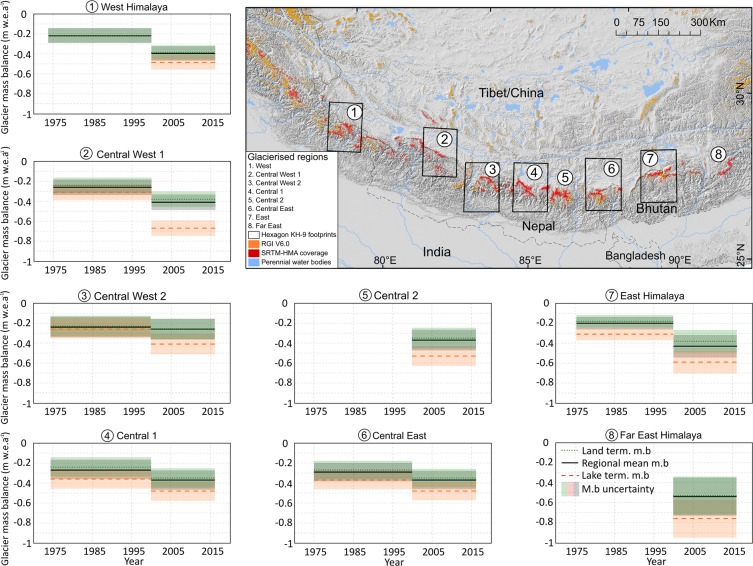
Regional glacier mass balance estimates across the Himalaya over the period ~1974–~2015, subdivided depending on glacier terminus type. Regional and land-terminating glacier mass balance estimates are the same for the period 2000–~2015 in Central West 1, and there are no lake-terminating glaciers covered in our ~1974–2000 dataset in the West Himalaya. Black boxes mark Hexagon footprint extent, which are lacking for Central 2 and Far East study areas due to inadequate quality of available Hexagon data. Orange polygons are from the Randolph Glacier Inventory version 6.0. Country boundaries are tentative and for orientation only. This figure was generated using ArcGIS, vers. 10.3 (http://www.esri.com/software/arcgis/arcgis-for-desktop) and Inkscape, vers. 0.92.4 (https://inkscape.org/).

We generated geodetic glacier mass balance estimates for two periods using digital elevation models (DEM) derived from Hexagon KH-9 stereoscopic imagery (spanning the period 1973–1976, supplementary information), the Shuttle Radar Topographic Mission DEM (2000), and 499 DEMs generated from WorldView and Geoeye optical stereo pairs (spanning the period 2012–2016^24^). Our assessment of contemporary (hereafter 2000–~2015) glacier mass loss rates covers a continuous swath from Jammu and Kashmir in the West of the Himalaya, to the Arunachal Pradesh in the Far East of the Himalaya (Fig. 1) and encompasses 1275 glaciers greater than 1 km^2^ in area (7450 km^2^ in total). Our assessment of 1973–6 to 2000 (hereafter ~1974–2000) glacier mass loss focusses on six regions (Fig. 1) and includes mass loss estimates for 939 glaciers (4834 km^2^ glacier area). We paired glacier mass balance data with estimates of glacier ice front retreat for a subset of 325 glaciers located in the same areas covered by Hexagon data. Ice front retreat was measured between the date of the Hexagon (1973–6), Landsat (1999–2002) and Sentinel imagery (2017/18).

## Results: Temporal Variability in Glacier Mass Loss

**Figure 2:**
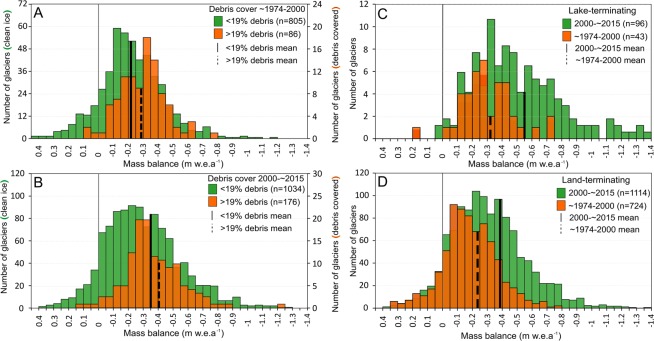
The distribution of glacier mass balance estimates for clean-ice (<19% debris cover) and debris-covered (>19% debris cover) glaciers over the period ~1974–2000 (**A**), and 2000–~2015 (**B**), lake-terminating (**C**) and land-terminating glaciers (**D**) across both study periods. Note variable scaling on y-axis.

Pervasive increases in ice mass loss and divergent ice mass loss depending on glacier terminus type are both evident in our results (Fig. 2). The mean mass balance of all glaciers within our sample over the period ~1974–2000 was −0.25 ± 0.09 m water equivalent (w.e) a^−1^, ranging from −0.20 ± 0.08 to −0.29 ± 0.10 m w.e.a^−1^. The mean mass balance of all glaciers between 2000 and ~2015 was −0.39 ± 0.12 m w.e.a^−1^, ranging from −0.26 ± 0.11 to −0.54 ± 0.20 m w.e.a^−1^ (Table 1). Glacier mass loss rates increased without exception in our study regions (Table 1, Fig. 1). Our results are in tendency in line with those of^9^, although our data do not support their finding that contemporary ice loss rates have increased to double those of the ~1974 to 2000 period (from −0.25 ± 0.09 to −0.39 ± 0.12 m w.e.a^−1^), as^9^ suggest (from −0.22 ± 0.13 to −0.43 ± 0.14 m w.e.a^−1^), particularly considering the levels of uncertainty associated with the mass balance data.

**Table 1:** Geodetic mass balance (mb) estimates for glaciers located in different regions across the Himalaya over the two time periods of this study.

Himalaya region	~1974 - to 2000				2000 to ~2015			
	Mean mb region (m w.e.a^−1^)	Mean mb lake-term. (m w.e.a^−1^)	Mean mb land-term. (m w.e.a^−1^)	Mean lake Vs land diff. (m w.e.a^−1^)	Mean mb region (m w.e.a^−1^)	Mean mb lake-term. (m w.e.a^−1^)	Mean mb land-term. (m w.e.a^−1^)	Mean lake Vs Land diff. (m w.e.a^−1^)
West	−0.21 ± 0.08	n/a	−0.21 ± 0.08	n/a	−0.40 ± 0.06	−0.49 ± 0.08	−0.39 ± 0.06	0.10
Central West 1	−0.26 ± 0.08	−0.31 ± 0.08	−0.24 ± 0.08	0.07	−0.41 ± 0.10	−0.67 ± 0.10	−0.38 ± 0.09	0.29
Central West 2	−0.24 ± 0.11	−0.26 ± 0.11	−0.23 ± 0.10	0.03	−0.26 ± 0.11	−0.41 ± 0.10	−0.26 ± 0.11	0.15
Central 1	−0.27 ± 0.10	−0.36 ± 0.10	−0.24 ± 0.10	0.12	−0.37 ± 0.11	−0.48 ± 0.12	−0.35 ± 0.11	0.13
Central 2	n/a	n/a	n/a	n/a	−0.37 ± 0.12	−0.53 ± 0.12	−0.35 ± 0.11	0.18
Central East	−0.29 ± 0.10	−0.37 ± 0.10	−0.27 ± 0.10	0.10	−0.37 ± 0.11	−0.48 ± 0.11	−0.35 ± 0.11	0.13
East	−0.20 ± 0.08	−0.31 ± 0.07	−0.18 ± 0.08	0.13	−0.43 ± 0.12	−0.59 ± 0.12	−0.38 ± 0.11	0.21
Far East	n/a	n/a	n/a	n/a	−0.54 ± 0.20	−0.76 ± 0.24	−0.53 ± 0.18	0.23
All	−0.25 ± 0.09	−0.32 ± 0.09	−0.23 ± 0.09	0.09	−0.39 ± 0.12	−0.55 ± 0.12	−0.37 ± 0.12	0.18

### The role of debris cover in glacier evolution

**Figure 3:**
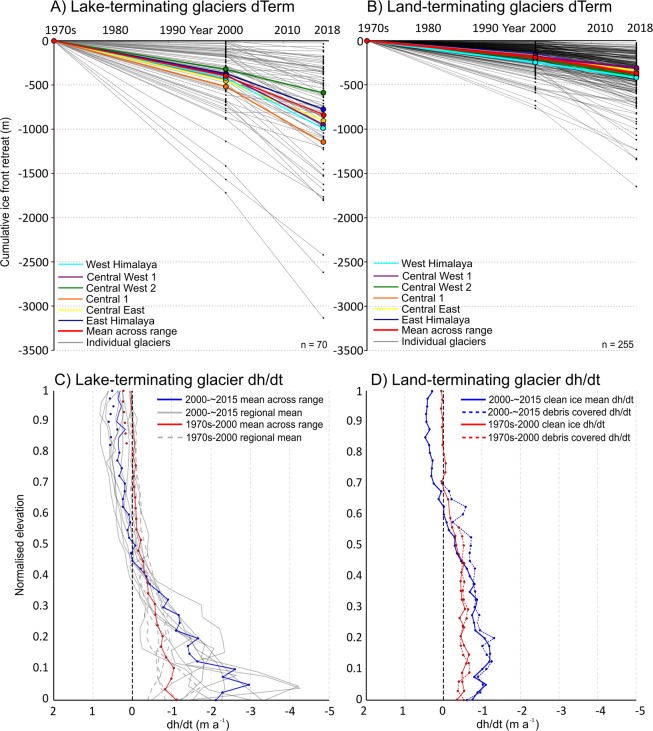
The cumulative ice front retreat (dTerm) of a subset (n = 325) of lake (**A**) and land-terminating (**B**) glaciers located across the Himalaya between 1973 and 2018. The relationship between glacier surface elevation change and elevation for lake-terminating (**C**) and land-terminating (**D**) glaciers with and without substantial debris cover.

To examine the relative importance of the presence of a debris mantle on glacier mass loss rates, we subdivided our mass balance datasets depending on debris extent, following the approach of^22^ (methods). Akin to^22^, we find no significant difference between thinning rates (Fig. 3) or mass balance of land-terminating glaciers with and without substantial debris cover. Over the period 2000–~2015 clean-ice, land-terminating glacier mass balance was −0.35 ± 0.12 m w.e.a^−1^, whereas debris-covered, land-terminating glacier mass balance was slightly more negative at −0.41 ± 0.12 m w.e.a^−1^. Further to^22^, we find similar mass loss rates irrespective of debris cover extent over the period ~1974–2000. Clean-ice, land-terminating glacier mass balance was −0.22 ± 0.08 m w.e.a^−1^, whereas debris-covered, land-terminating glacier mass balance was again slightly more negative at −0.29 ± 0.08 m w.e.a^−1^. These results show that similar ice loss from debris-covered compared to debris-free glaciers is not a recent phenomenon. Using unpaired, two-tailed t-tests, we examined the statistical characteristics of the differences between mass balance estimates for debris-covered and clean-ice glaciers (supplementary tables 7 and 8). In five of our eight sub-regions, we find little evidence of significant differences in the mass balance of debris-covered and clean-ice glaciers (p > 0.05, t 0.05–1.35) over the period 2000–~2015 (supplementary table 8). The coverage of our ~1974–2000 mass balance dataset did not allow for the statistical analyses of differences in all sub-regions, but in four cases we find little evidence of significant differences in the mass balance of debris-covered and clean-ice glaciers (p > 0.05, t 1.07–1.91) over this study period.

### Terminus type variability in ice loss

The mean mass balance of lake-terminating glaciers was substantially more negative than that of land-terminating glaciers (Table 1), thus we focus the remainder of our analyses on the impact of glacier-lake interactions on glacier mass loss. Over the period ~1974–2000, lake-terminating glacier mass balance (mean −0.32 ± 0.12 m w.e.a^−1^) more negative than land-terminating glacier mass balance (−0.23 ± 0.09 m w.e.a^−1^) across the Himalaya (Fig. 2), ranging from 0.03 m w.e.a^−1^ (Central West 1) to 0.13 m w.e.a^−1^ (East) for specific regions (Table 1). Over the period 2000–~2015, the difference between lake-terminating glacier mass balance (−0.55 ± 0.12 m w.e.a^−1^) and land-terminating glacier mass balance (−0.37 ± 0.12 m w.e.a^−1^) was double that of the earlier time period, again varying from 0.10 m w.e.a^1^ (West) to 0.29 m w.e.a^1^ (Central West 1) for different sub-regions (Table 1). The mass balance of debris-covered (−0.51 ± 0.12 m w.e.a^−1^) and clean-ice (−0.67 ± 0.15 m w.e.a^−1^) lake-terminating glaciers were both substantially more negative than land-terminating, debris-covered (−0.41 ± 0.12 m w.e.a^−1^) and clean-ice glaciers (−0.35 ± 0.12 m w.e.a^−1^) (supplementary table 4), thus terminus type appears to exert a much stronger influence on glacier mass balance than debris extent.

Again we examined the statistical characteristics of terminus-type dependant differences in our mass balance datasets (supplementary tables 7 and 8). In two out of three sub-regions tested for the period ~1974–2000 p < 0.05, although t-values were low (2.05–2.51), suggesting a less robust relationship between terminus type and mass loss rates over this earlier period. In the five sub-regions where data quantity allowed for statistical analyses, terminus type dependant differences in mass balance were all significant (p < 0.05, t 2.65–5.88) over the period 2000–~2015, which suggests the much greater impact of glacial lake growth on glacier mass loss rates towards the present day over large parts of the Himalaya.

Glacier terminus retreat accompanied the widespread glacier thinning across the Himalaya (Figure 3). Over the period ~1974–2000, land-terminating glaciers retreated at a mean rate of 7.1 ± 1.1 m a^−1^, ranging only slightly between regions (Supplementary Table 6). Lake-terminating glaciers retreated at a mean rate of 15.9 ± 1.1 m a^−1^ over the same period. Glacier terminus retreat rates increased without exception across the two time periods, to a mean rate of 10.4 ± 1.4 m a^−1^ for land-terminating glaciers and 26.8 ± 1.4 m a^−1^ for lake-terminating glaciers, respectively, over the period 2000–2018 (Supplementary Table 5). The retreat rate of land-terminating glaciers increased on average by ~46% between the two study periods, whereas lake-terminating glacier retreat rates increased by almost 70%. Along glacier centrelines (see methods), land-terminating glaciers reduced in length by a mean value of 9%, ranging from no change (where heavily debris**-**covered) to 33%, between the 1970s and 2018. Lake-terminating glacier length reduced by a mean of 13%, ranging from <1 to 49%, over the same period.

Examination of the altitudinal distribution of glacier surface elevation changes shows ice loss at the glacier-lake interface to be the main driver of the enhanced mass loss from lake-terminating glaciers (Fig. 3). Thinning rates of ~1 m a^−1^ were pervasive for ablation zones of land-terminating glaciers across the Himalaya (Fig. 3) over the period 2000–~2015. In contrast, lake-terminating glaciers thinned by up to 4 m a^−1^ at their termini in some regions (Eastern Himalaya), and large portions of their ablation zones thinned at a greater rate than land-terminating glaciers. Similar thinning patterns are evident for glaciers of different terminus type over the period ~1974–2000 (Fig. 3), although thinning rates were of lesser magnitude. Land-terminating glacier ablation zones thinned at a rate of ~0.5 m a^−1^ over the period ~1974–2000, whereas lake-terminating glacier ablation zones lowered at a mean rate of ~1 m a^−1^ over the same period.

Lake-terminating glaciers constituted only a small portion of the glacier population in each region, yet they were responsible for a substantial amount of the regional ice mass loss, across both study periods (Table 2). Lake-terminating glaciers accounted for ~32% of the ice mass loss in our Central West 1 study area (Fig. 1) over the period ~1974–2000, despite just ~9% of the glacier population terminating into a lake. Lake-terminating glaciers in the Central 1, the Central East and East Himalaya contributed ~20% of the total regional ice mass loss whilst accounting for 11–14% of the glacier population over the period ~1974–2000. The contribution of lake-terminating glaciers to intra-regional ice mass loss budgets increased by ~21% after 2000, where glacial lakes are prevalent. Lake-terminating glaciers in Central West 1, Central East and East Himalaya provided similar proportions (30, 30 and 29%, respectively) of the total regional mass loss over this period (Table 2). The regional mass balance in the Central West 2 region, where only a few lake-terminating glaciers are situated, remained almost unchanged (−0.24 ± 0.11 Vs −0.26 ± 0.11 m w.e. a^−1^) between the two study periods.^9^ estimated that only 5–6% of the total ice mass loss from the entire Himalaya is provided by lake-terminating glaciers, although their analyses is limited to glaciers >3 km^2^ in size, and^9^ show that smaller lake terminating glaciers generally display the most negative mass balance.

**Table 2:** The contribution of lake-terminating glaciers to glacier mass loss from different catchments over the two time periods of this study.

Himalaya Region	~1974 to 2000		2000 to 2013–16	
	% of glacier population lake-terminating glaciers	% contribution of regional ice mass loss	% of glacier population lake-terminating glaciers	% contribution of regional ice mass loss
West	n/a	n/a	<1	1
Central West 1	9	32	10	30
Central West 2	1	4	1	4
Central 1	11	17	14	23
Central 2	n/a	n/a	9	15
Central East	11	20	15	30
East	14	21	22	29
Far East	n/a	n/a	4	11
All	9.2 (11)	18.8 (23)	9.5(15)	18(28)

Numbers in parentheses represent mean values for the West, Central 1, Central East and East Himalaya regions, where glacial lakes are most prevalent.

We measured comparable mass loss rates from glaciers in the (West) Himalaya, where few glacial lakes are situated, to regions where glacial lakes have exacerbated ice mass loss (Table 1). Glaciers in Garwhal Himalaya exist in a unique climatological setting. They receive the majority of their precipitation from mid-latitude winter westerlies^1,25^, but experience mean annual temperatures more akin to the central Himalaya, rather than the colder Karakoram^8^. The sensitivity of snowfall to warming is therefore higher in this region, and long-term temperature increases^8,26^ have heavily impacted both seasonal snowfall^26,27^ the phase of summer precipitation^17,28^, and therefore glacier mass balance in this region.

## Discussion: Implications for Future Glacier Evolution

Our results clearly emphasise the strong impact of glacial lake development on glacier recession along the Himalaya since the mid-1970s, alongside atmospheric warming^9^. Over this period, lake-terminating glacier mass balance was substantially more negative than that of land-terminating glaciers, and lake-terminating glacier termini retreated at twice the rate of their land-terminating counterparts. Although lake-terminating glaciers make up only a small portion of the total glacier population (~10%), they are responsible for a disproportionate share of intra-regional ice mass loss. Where lake-terminating glaciers are most prevalent (Central West 1, Central 1, Central East and East Himalaya), lake-terminating glacier recession accounted for almost 30% of the total ice mass loss, despite comprising only ~15% of the glacier population, over the period 2000–~2015. This contribution increased from ~23% over the period ~1974–2000, when ~11% of the glacier population terminated into glacial lakes. Statistical analyses of our mass balance datasets also indicate the now widespread influence of glacier terminus type on glacier mass loss rates. Where glacial lakes were not prevalent (Central West 2), regional mass loss rates have remained steady over the last four decades.

The magnitude of the contribution of lake-terminating glaciers to regional ice loss is unlikely to diminish in coming decades, given the sustained expansion of currently proglacial lakes across the Himalaya^11,20,29^, and the preconditioning of many debris-covered, land-terminating glacier surfaces for meltwater storage.^30^ suggest that the transition of many debris-covered glaciers from land-terminating to lake-terminating is a likely scenario in the later stages of glacier wastage. Indeed, more than 25% of the debris-covered glaciers we examined hosted glacial lakes, and debris-covered, lake-terminating glaciers displayed the highest mass loss rates of all glaciers we surveyed (−0.67 ± 0.15 m w.e.a^−1^, supplementary table 4). Widespread glacier surface velocity reductions^31^, sustained glacier thinning (Fig. 3) and associated surface slope reductions^32^ will allow for the formation of more extensive supraglacial pond networks on many debris**-**covered glaciers, which will eventually coalesce to become pro-glacial lakes^31^. The heightened mass loss from such glaciers will sustain their contribution to the regional mass loss budget in coming decades.

Our results show that several decades of enhanced ice loss is possible whilst glacier-lake interactions drive the dynamic evolution of such glaciers. Increased thinning rates and amplified terminus retreat rates (Fig. 3) were documented for the majority of the population of lake-terminating glaciers we assessed over the >40 year study period. The amplified thinning towards lake-terminating termini is due to the occurrence of both mechanical calving and subaqueous melt^18,19^. The increase in thinning rates over lake-terminating glaciers across the two study periods (Fig. 3) is likely to have been driven by the increased areal extent^11,29^ and the depth^33^ of glacial lakes across the region in recent decades. Increased proglacial lake depth exacerbates calving fluxes^18,34^ and increases the glacier-lake contact area prone to subaqueous melt and can also influence glacier flow rates^32^, which increases ice fluxes towards the lake each glacier hosts. The dynamic behaviour of lake-terminating glaciers is in stark contrast to land-terminating glaciers along the Himalaya, which have experienced substantial velocity reductions in response to thinning and driving stress reductions since 2000^31^.

The comparability of ice loss rates from debris-covered and clean-ice glaciers suggests that localised ablative processes, such as ice cliff and supraglacial pond expansion^35–37^, have contributed substantially to individual glacier mass budgets for much longer than previously thought, even during times of less negative glacier mass balance. Estimates of the contribution of ice-cliff backwasting to individual glacier ablation budgets in the Himalaya range from 7–40%^36–38^. suggest that the absorption and redistribution of energy by supraglacial ponds may account for 6–19% of surface ablation on debris**-**covered glaciers in the Langtang catchment. In combination, these processes may drive substantial ablation in heavily debris-mantled areas of glaciers. Pervasive glacier stagnation^31^ may also be contributing to the comparability of debris**-**covered and clean-ice glacier thinning rates, with reduced emergence velocities in debris**-**covered areas^38^ aiding thinning. Disentangling the contribution of each ablative process is key to understanding the evolution of debris-covered, land-terminating glaciers in the Himalaya.

In order to understand whether the contribution of lake-terminating glaciers to regional ice mass loss may increase further, both the prevalence of the formation of new glacial lakes, and the impact of multi-decadal glacier thinning on the dynamics of lake-terminating glaciers need to be better understood. If lake-terminating glacier behaviour is not considered in future ice mass loss scenarios, ice mass loss from the Himalaya, and other regions where glacial lakes are common, may be substantially underestimated.

## Methods

### DEM pre-processing and dh/dt correction

The methods of^39^ were followed to eliminate planimetric and altimetric shifts from HMA DEMs and Hexagon KH-9 DEMs. The non void-filled, 30 m resolution SRTM DEM (https://earthexplorer.usgs.gov/) was used as the reference DEM and the RGI V6.0 glacier inventory^40^, which was modified manually to reflect glacier extent visible in the Hexagon imagery from the 1970s, was used to isolate dh/dt data over stable ground from which shift vectors were calculated. Along-track and cross-track biases were not prevalent in HMA DEMs. To remove tilts from Hexagon KH-9 DEMs, a second order global trend surface was fitted to non-glacierised terrain, considering elevation differences between ±150 m and inclination ≤15°^28^. Following the coregistration of DEMs from different epochs, individual DEMs were differenced to obtain elevation change data over different time periods.

The SRTM DEM is known to have underestimated glacier surface elevations due to C-band radar penetration^41^. Failure to correct such a penetration bias may cause a 20% underestimate in regional mass balance estimates^42^. We corrected dh/dt data derived using the SRTM DEM using the penetration estimates of^15^, which were estimated through the reconstruction of glacier surface elevations at the point of SRTM acquisition via the extrapolation of a time series of IceSat data (spanning the period 2003–2009), with the difference between the two datasets assumed to represent C-band penetration depths. The direct validation of SRTM penetration depth estimates are difficult due to the lack of information available about spatially variable glacier surface conditions (snowpack depth and extent) at the time of SRTM DEM acquisition. We compared our geodetic mass balance estimates with those derived using alternative methods and baseline datasets not affected by C-band radar penetration (Supplementary Table 3), and find a mean difference of −0.02 m w.e. a^−1^ (ranging from −0.12 to + 0.08 m w.e. a^−1^) between estimates of regional mass loss over directly comparable time periods generated by^6^. This suggests the successful elimination of C-band radar penetration biases.

The derivation of geodetic mass balance estimates involves the summation of glacier mass loss or gain over the entirety of a glacier’s surface. Variable glacier surface conditions and the extreme topography of glacierised mountain regions means data gaps and anomalous surface elevation values are common in DEMs generated from remotely-sensed imagery. Data gaps and anomalies are inherited by glacier surface elevation change data once DEMs from two time periods are differenced, and they must be filled or removed through filtering for glacier mass loss to be captured accurately.

The approach of^43^ was employed to filter the surface elevation change data generated using Hexagon KH-9 data. This approach involves the filtering of surface elevation change data depending on the standard deviation of elevation changes, weighted by an elevation dependent coefficient. The approach of^43^ allows for stricter filtering of elevation change data at higher elevations, where outliers arising from poor image contrast in glacier accumulation zones are common and where the magnitude of elevation changes are expected to be lower. More lenient filtering of elevation change data is required over glacier ablation zones, where optical contrast and therefore Hexagon DEM quality was higher.

The improved spatial and spectral resolution of the WorldView and Geoeye imagery in comparison to the Hexagon data means superior coverage of DEMs was available over glacier accumulation zones in our later study period. The remnant anomalies present in our contemporary (SRTM-HMA) surface elevation change dataset, mainly resulting from errors in the SRTM DEM, were eliminated following the simpler approach of^44^. The approach of^44^ involves the removal of values greater than +/− 3 standard deviations of the mean elevation change in 100 m altitudinal bins through the elevation range of glacierised terrain.

We employed a two-step gap filling approach; first we used a 4 × 4 cell moving window to fill small (a few pixels) data gaps with mean elevation change data from neighbouring cells. We then filled larger data gaps with median values of surface elevation change calculated across each 100 m increment of the glaciers elevation range. Both approaches have been shown to have limited impact on glacier mass loss estimation^45^. Data gaps were most prevalent in surface elevation change data derived from Hexagon imagery, varying from 5.5–14.5% of glacier area for different sub-regions. We converted surface elevation change data to ice volume considering the grid size of our dh/dt data (30 m pixels), and then to glacier mass change using a conversion factor of 850 ± 60 kg m^−3^ ^46^.

### Glacier mass balance subdivision

We divided our samples of glacier mass balance depending on their terminus type and debris extent. Terminus type was determined manually using satellite imagery from each date as reference, with contact required between a proglacial lake and its host glacier to allow for its classification of lake-terminating. We replicated the approach of^22^ to divide our mass balance data depending on debris-extent, and classified glaciers as debris**-**covered where more than 19% of their area was mantled by debris, and as clean-ice otherwise, using the supraglacial classification of^10^.

### Mass balance uncertainty

Our mass balance uncertainty (σ_Δm_) estimates consider and combine the uncertainty associated with surface elevation change (E_Δ*h*_), the uncertainty associated with volume to mass conversion (*E*_Δ*m*_), and the spatially nonuniform distribution of uncertainty.

The uncertainty associated with elevation change (*E*_Δ*h*_) was calculated through the derivation of the standard error - the standard deviation of the mean elevation change - of 100 m altitudinal bands of elevation difference data^35,45^:

$${E}_{\varDelta h}=\frac{{\sigma }_{stable}}{\sqrt{{\rm{N}}}}$$

Where *σ*_*stable*_ is the standard deviation of the mean elevation change of stable, off-glacier terrain, and *N* is the effective number of observations^46^. *N* is calculated through:

$$N=\frac{{N}_{tot}\cdot PS\,}{2d}$$

Where *N*_*tot*_ is the total number of DEM difference data points, *PS* is the pixel size and *d* is the distance of spatial autocorrelation, taken here to equal 20 pixels (600 m). *E*_Δ*m*_ was calculated as 7% of the mass loss estimate^47^ for each glacier and summed quadratically with *E*_Δ*h*_:

$${{\rm{\sigma }}}_{\varDelta {\rm{m}}}=\sqrt{{{\rm{E}}}_{\varDelta {\rm{h}}}^{2}+{E}_{\varDelta m}\,}$$

σ_Δm_ was then weighted depending on glacier hypsometry in each region to better represent the spatial variability of uncertainty^35^.

### Glacier terminus mapping

Glacier termini were mapped for three different epochs using the same six Hexagon KH-9 scenes used in DEM generation, 7 Landsat TM/ETM+ scenes spanning the period 1999–2002, and 6 Sentinel 2 A/B scenes spanning the period 2016 to 2018 (Supplementary Table 2). We also used 8 orthorectified Corona KH-4B images analysed by^48^ to map glacier termini in Himachal Pradesh (West Himalaya). Glacier termini were mapped in a semi-automated fashion using the approach of^49^, which involves the manual digitisation of glacier termini, the division of the ice front into points of even spacing, and the measurement of the distance between terminus points to a reference location placed up glacier. We generated glacier centreline profiles for the extent of glaciers in the Hexagon imagery following the approach of^50^ to quantify the impact of terminus retreat on glacier length over the study period.

### Glacier terminus change uncertainty

We followed the approach of^51^ to estimate the uncertainty associated with terminus retreat rates, whereby:

$$e=\sqrt{{({\rm{PS}}1)}^{2}+{({\rm{PS}}2)}^{2}}+{{\rm{E}}}_{{\rm{reg}}}$$

Where *e* is the total error in terminus position, PS1 is the pixel size of imagery from the first epoch, PS2 is the pixel size of imagery from the second epoch, and E_reg_ the coregistration error between images, which we assume to be half a pixel^52^.

## Supplementary information

Supplementary Information

## References

[1] Bolch T (2012). The state and fate of Himlayan glaciers. Science.

[2] Farinotti Daniel, Longuevergne Laurent, Moholdt Geir, Duethmann Doris, Mölg Thomas, Bolch Tobias, Vorogushyn Sergiy, Güntner Andreas (2015). Substantial glacier mass loss in the Tien Shan over the past 50 years. Nature Geoscience.

[3] Farinotti D (2015). Substnatial glacier mass loss in the Tien Shan over the past 50 years. Nat. Geosci..

[4] Zhou Y, Li Z, Li J, Zhao R, Ding X (2018). Glacier mass balance in the Qinghai-Tibet Plateau and its surroundings from the mid 1970s to 2000 based on Hexagon KH-9 and SRTM DEMs. Rem. Sens. Environ..

[5] Kääb A, Triechler D, Nuth C, Berthier E (2015). Contending estimates of 2003–2008 glacier mass balance over the Pamir-Karakoram-Himalaya. Cryosphere..

[6] Brun F, Berthier E, Wagnon P, Kääb A, Treichler D (2017). A spatially resolved estimate of High Mountain Asia glacier mass balances from 2000 to 2016. Nat. Geosci..

[7] Bolch T, Pieczonka T, Mukherjee K, Shea J (2017). Glaciers in the Hunza catchment (Karakoram) have been nearly in balance since the 1970s. Cryosphere.

[8] Kapnick SB, Delworth TL, Ashfaq M, Malyshev S, Milly PCD (2014). Snowfall less sensitive to warming in the Karakoram than in Himalayas due to a unique seasonal cycle. Nature..

[9] Maurer, J., Schaefer, J. M., Rupper, S. & Corley, A. Acceleration of ice loss across the Himalayas over the past 40 years. *Sci. Adv*., **5** (2019). 10.1126/sciadv.aav7266 PMC6584665 31223649

[10] Kraaijenbrink, P. D. A., Bierkens, M. F. P., Lutz, A. F. & Immerzeel, W. W. Impact of a global temperature rise of 1.5 degrees Celsius on Asia’s glaciers. *Nature*, **549** (2017). 10.1038/nature23878 28905897

[11] Nie Y (2017). A regional-scale assessment of Himalayan glacial lake changes using satellite observations from 1990–2015. Remote Sense. Environ..

[12] Nicholson L, Benn DI (2006). Calculating ice melt beneath a debris layer using meteorological data. J. Glacio..

[13] Anderson LS, Anderson RS (2016). Modeling debris-covered glaciers: responds to steady debris deposition. Cryosphere.

[14] Rowan AV, Egholm DL, Quincey DJ, Glasser NF (2015). Modelling the feedbacks between mass balance, ice flow and debris transport to predict the response to climate change of debris-covered glaciers in the Himalaya. Earth. Plan. Sci. Lett..

[15] Kääb A, Berthier E, Nuth C, Gardelle J, Arnaud Y (2012). Contrasting patterns of early twenty-first-century glacier mass change in the Himalayas. Nature.

[16] Nuimura T, Fujita K, Yamaguchi S, Sharma RR (2012). Elevation changes of glaciers revealed by multitemporal digital elevation models calibrated by GPS survey in the Khumbu region, Nepal Himalaya, 1992–2008. J. Glacio..

[17] Pratap, B., Dobhal, D. P., Mehta, M. & Bhambri, R. Influence of debris cover and altitude on glacier surface melting: a case study on Dokriani Glacier, central Himalaya, India. *Ann. Glacio*., **56** (2015).

[18] Benn DI, Warren CR, Mottram RH (2007). Calving processes and the dynamics of calving glaciers. Earth. Sci. Rev..

[19] Truffer M, Motyka RJ (2016). Where glaciers meet water: Subaqueous melt and its relevance to glaciers in various settings. Rev. Geophys..

[20] Zhang G (2015). An inventory of glacial lakes in the Third Pole region and their changes in response to global warming. Global and Planetary Change.

[21] Basnett S, Kulkarni A, Bolch T (2013). The influence of debris cover and glacial lakes on the recession of glaciers in Sikkim Himalaya, India. J. Glaciol..

[22] Brun, F. *et al*. Heterogeneous influence of glacier morphology on the mass balance variability in High Mountain Asia. J. Geophys. Res. Earth. Surf., (2019).

[23] Song C (2017). Heterogeneous glacial lake changes and links of lake expansions to the rapid thinning of adjacent glacier termini in the Himalayas. Geomorphology.

[24] Shean DE (2016). An automated, open-source pipeline for mass production of digital elevation models (DEMs) from very-high-resolution commercial stereo satellite imagery. J.Photogram. Remote. Sens..

[25] Vijay S, Braun M (2018). Early 21^st^ century spatially detailed elevation changes of Jammu and Kashmir glaciers (Karakoram-Himalaya). Glob. Plan. Change..

[26] Shekhar MS, Chand H, Kumar S, Srinivasan K, Ganju A (2010). Climate-change studies in the western Himalaya. Ann. Glacio..

[27] Yao T (2012). Different glacier status with atmospheric circulations in Tibetan Plateau and surroundings. Nature Clim. Change..

[28] Pieczonka T, Bolch T, Wie J, Liu S (2013). Heterogeneous mass loss of glaciers in the Aksu-Tarim Catchment (Central Tien Shan) revealed by 1976 KH-9 Hexagon and 2009 SPOT-5 stereo imagery. Remote Sen. Environ..

[29] Khadka, N., Zhang, G. & Thakuri, S. Glacial Lakes in the Nepal Himalaya: Inventory and Decadal Dynamics (1977–2017). *Remote Sens*., **10** (2018).

[30] Benn D (2012). Response of debris-covered glaciers in the Mount Everest region to recent warming, and implications for outburst flood hazards. Earth-Sci. Rev..

[31] Dehecq A (2019). Twenty-first century glacier slowdown driven by mass loss in High Mountain Asia. Nat. Geosci..

[32] King O, Dehecq A, Quincey DJ, Carrivick JL (2018). Contrasting geometric and dynamic evolution of lake and land-terminating glaciers in the central Himalaya. Glob. Plan. Change..

[33] Somos-Valenzuela MA, McKinney DC, Rounce DR, Byers AC (2014). Changes in Imja Tsho in the Mount Everest region of Nepal. Cryosphere..

[34] Kirkbride M, Warren CR (1997). Calving processes at a grounded ice cliff. Ann. Glacio..

[35] Ragettli S, Bolch T, Pellicciotti F (2016). Heterogeneous glacier thinning patterns over the last 40 years in Langtang Himal, Nepal. Cryosphere..

[36] Thompson S, Benn DI, Mertes J, Luckman A (2016). Stagnation and mass loss on a Himalayan debris-covered glacier: processes, patterns and rates. J. Glacio..

[37] Miles ES (2018). Surface Pond Energy Absorption Across Four Himalayan Glaciers Accounts for 1/8 of Total Catchment Ice Loss. Geophys. Res. Lett..

[38] Brun F (2018). Ice cliff contribution to the tongue-wide ablation of Changri Nup Glacier, Nepal, central Himalaya. Cryosphere..

[39] Nuth C, Kääb A (2011). Co-registration and bias corrections of satellite elevation data sets for quantifying glacier thickness change. Cryosphere..

[40] RGI Consortium. Randolph Glacier Inventory – A Dataset of Global Glacier Outlines: Version 6.0: Technical Report, Global Land Ice Measurements from Space, Colorado, USA. Digital Media (2017).

[41] Gardelle J, Berthier E, Arnaud Y (2012). Impact of resolution and radar penetration on glacier elevation changes computed from DEM differencing. J. Glacio..

[42] Vijay, S. & Braun, M. Elevation change rates of glaciers in the Lahaul-Spiti (Western Himalaya, India) during 2000–2012 and 2012–2013. *Rem. Sens*. **8** (2016).

[43] Pieczonka T, Bolch T (2015). Region-wide glacier mass budgets and area changes for the Central Tien Shan between ~1975 and 1999 using Hexagon KH-9 imagery. Glob. Plan. Change..

[44] Gardelle J, Berthier E, Arnaud Y, Kääb A (2013). Region-wide glacier mass balances over the Pamir-Karakoram-Himalaya during 1999–2011. Cryosphere.

[45] McNabb R, Nuth C, Kääb A, Girod L (2019). Sensitivity of glacier volume change estimation to DEM void interpolation. Cryosphere.

[46] Bolch T, Pieczonka T, Benn DI (2011). Multi-decadal mass loss of glaciers in the Everest area (Nepal Himalaya) derived from stereo imagery. Cryosphere.

[47] Huss M (2013). Density assumptions for converting geodetic glacier volume change to mass change. Cryosphere.

[48] Mukherjee K, Bhattacharya A, Pieczonka T, Ghosh S, Bolch T (2018). Glacier mass budget and climatic reanalysis data indicate a climate shift around 2000 in Lahaul-Spiti, western Himalaya. Climatic Change..

[49] Bjørk AA (2012). An aerial view of 80 years of climate-related glacier fluctuations in southeast Greenland. Nat. Geosci..

[50] James WHM, Carrivick JL (2016). Automated modelling of spatially-distributed glacier ice thickness and volume. Comp. & Geosci..

[51] Hall DK, Bayr KJ, Schöner W, Bindschadler RA, Chien JY (2003). Consideration of the errors inherent in mapping historical glacier positions in Austria from the ground and space (1893–2001). Remote Sens. Environ..

[52] Bolch T (2010). A glacier inventory for the western Nyainqentanglha Range and the Nam Co Basin, Tibet, and glacier changes 1976–2009. Cryosphere.

