# Supplementary material for: Glacial lakes exacerbate Himalayan glacier mass loss
Source: Sci Rep. 2019 Dec 2;9:18145. doi: 10.1038/s41598-019-53733-x (PMC6889251; doi:10.1038/s41598-019-53733-x)
Supplement: Supplementary file 1 — Supplementary Information [file 41598_2019_53733_MOESM1_ESM.docx]

**Supplementary information**

**Regional subdivision**

We present mass balance data at a more localised scale than previous studies to avoid the masking of the spatial variability of mass loss through regional averaging. The region named as the West Himalaya includes glaciers in Jammu and Kashmir, the Himachal Pradesh and Uttarakhand in North Western India. The region named Central West 1 covers the westernmost part of Nepal and a section of the Nepal- Tibet/China border from which glaciers flow north onto the Tibetan Plateau. The region named Central West 2 includes glaciers proximal to the 8000m peaks Annapurna I and Manasalu. The region named Central 1 includes glaciers in the Langtang National park in Nepal and the Poiqu river basin in Tibet/China. The region named Central 2 covers the Sagarmatha national park in Nepal and glaciers flowing onto the Tibetan Plateau to the North of Mt. Everest. The region named Central East Himalaya covers the Sikkim region of India and glaciers flowing North onto the Tibetan Plateau. The region named East Himalaya covers Bhutan. The region named Far East Himalaya covers glaciers in the Arunachal Pradesh (India) and glaciers flowing north onto the Tibetan Plateau. The extent of all subregions is shown in Figure S1.

**Coverage of mass balance dataset**

Our assessment of glacier mass loss is centred on the main Himalayan arc because of the prevalence of lake-terminating glaciers here. We used 499 of the DEMs produced by the Polar Geospatial Centre (University of Minnesota) from a portion of the WorldView 1, WorldView 2 and Geoeye archives spanning the period 2012-2016. In combination with the SRTM DEM, we derived mass balance estimates for 1275 glaciers (7450 km^2^ glacier area) between the Himachal Pradesh in the West, and the Arunchal Pradesh in the East. The coverage of the publically available HMA DEMs (Shean, 2017) is not sufficient to provide a spatially resolved estimate of glacier mass loss, such as that produced by Brun et al. (2018), but we assume that the achievable coverage of geodetic mass balance data is representative of the whole glacier population in each region.

**Supplementary Table 1**. Summary of glacier mass balance dataset coverage over the two time periods. % cover relates to glacier area within the sub-regions defined in Figure S1.

|  | **1974-2000** |  |  | **2000-~2016** |  |  |
| --- | --- | --- | --- | --- | --- | --- |
| **Region** | **N mb estimates** | **N glaciers** | **Glacier area (km^2^)/ % cover** | **N mb estimates** | **N glaciers** | **Glacier area (km^2^)/ % cover** |
| West | 172 | 172 | 1095/13 | 432 | 356 | 2527/30 |
| Central West 1 | 127 | 127 | 540/36 | 216 | 138 | 682/46 |
| Central West 2 | 182 | 182 | 744/45 | 368 | 191 | 871/53 |
| Central 1 | 148 | 148 | 840/71 | 189 | 138 | 829/71 |
| Central 2 | - | - | - | 326 | 125 | 979/57 |
| Central East | 169 | 169 | 746/62 | 288 | 128 | 600/50 |
| East | 141 | 141 | 869/91 | 222 | 148 | 746/79 |
| Far East | - | - | - | 81 | 51 | 216/49 |
| All | 939 | 939 | 4834/53 | 2057 | 1275 | 7450/54 |

The repetitive acquisition of the WorldView and Geoeye satellites over the period covered by the HMA DEMs means that a large portion of glacier area is covered by more than one DEM acquisition (Shean et al., 2016) along the main Himalayan arc. Such data abundance allowed for the derivation of glacier mass balance estimates over a combination of different time periods (although still between a HMA DEM and the SRTM DEM). Where more than one mass balance estimate was available for a glacier, we use the mean value of mass loss. Such an approach minimises the impact of seasonal variability on glacier mass balance estimates.


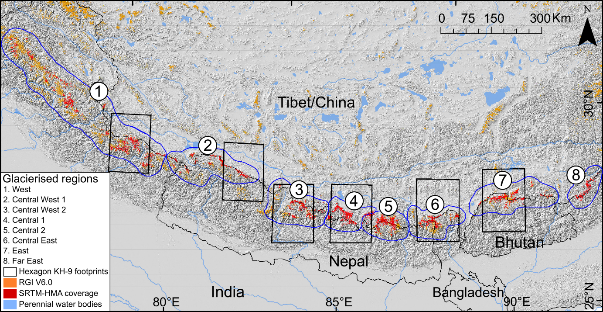


**Figure S1**. The coverage of dhdt data generated in this study. This figure was generated using ArcGIS, vers. 10.3 (www.esri.com/software/arcgis/arcgis-for-desktop). Used data sources: SRTM DEM (https://earthexplorer.usgs.gov/), Randolph Glacier Inventory, vers. 6.0 (RGI Consortium, 2017, www.glims.org/RGI), Global River Network (GRIN, Schneider et al., 2017, www.metis.upmc.fr/fr/node/375), Global Lakes and Wetlands Database (GWLD, https://www.worldwildlife.org/pages/global-lakes-and-wetlands-database).

Our assessment of pre-millennium glacier mass loss is more spatially confined due to the limited availability of Hexagon imagery, which was acquired in a more sporadic manner than modern day stereo imagery (Surazakov and Aizen 2010). Sets of Hexagon imagery acquired between 1973 and 1976 were selected which covered six areas of the Himalayan arc (Figure S1). We did not find Hexagon imagery with suitable glacier surface conditions, due to the presence of cloud or snow cover, to allow for the generation of DEMs from the 1970s for the Central 2 region or Far East Himalaya.

**Prevalence of gap filling in Hexagon data**

A commonly encountered limitation of the use of declassified Hexagon imagery in DEM generation is the limited contrast available in imagery over areas of similar spectral characteristics. In glacierised terrain, such surface conditions are common in glacier accumulation zones, where snow cover presents a homogenous surface. Spurious surface elevation data may result in DEMs over such areas and large data gaps may be present in dh/dt data, following filtering. Incomplete data coverage over the higher portions of glacier elevation ranges could lead to the underestimation of accumulation, and therefore bias towards more negative mass balance estimates. Careful image selection is therefore vital to limit the prevalence of data gaps, and robust filtering and gap filling methods are required to preserve the correct signal of elevation change in glacier accumulation zones.

The portion of dh/dt data that required gap filling varied between different regions but was limited to 5.5-14.5% of glacier area for dh/dt data generated using Hexagon imagery (Supplementary Table 2). Data gaps were much less prevalent in the SRTM-HMA DEM dataset. Supplementary Table 2. Percentage of dh/dt over glacier area where outlier filtering caused data gaps in dh/dt data derived from Hexagon imagery for each sub-region.

**Supplementary Table 2.** Prevalence of data voids in ~1974-2000 dh/dt data for different sub-regions covered by Hexagon imagery.

| Sub-region | % glacier area composed of data voids |
| --- | --- |
| West | 13.2 |
| Central West 1 | 14.5 |
| Central West 2 | 5.6 |
| Central 1 | 9.8 |
| Central East | 10.4 |
| East | 11.2 |

**Supplementary Table 3.** Imagery used in glacier terminus mapping (1) and DEM generation (2).

| Himalayan region | Sensor | Date | Scene ID | Data usage |
| --- | --- | --- | --- | --- |
| East | Hexagon KH9 | 07/01/1976 | DZB1211-500125L014001 | 1,2 |
|  | Hexagon KH9 |  | DZB1211-500125L015001 | 1,2 |
|  | Landsat 5 | 09/11/2000 | LT51380402000314BKT00 | 1 |
|  | Sentinel-2A | 21/12/2017 | L1C_T45RYM_A013043_20171221T044710 | 1 |
|  |  | 04/02/2018 | L1C_T46RBS_A004778_20180204T044709 | 1 |
| Central 1 | Hexagon KH9 | 23/11/1974 | DZB1209-500101L006001 | 1,2 |
|  | Hexagon KH9 |  | DZB1209-500101L007001 | 1,2 |
|  | Landsat 5 | 13/10/2000 | LT51410402000287BKT00 | 1 |
|  | Sentinel-2A | 25/09/2018 | L1C_T45RUM_A008110_20180925T045829 | 1 |
|  |  | 18/10/2018 | L1C_T45RUM_A008439_20181018T045821 | 1 |
| West | Corona (KH4B) | 28.09.1971 | DS1115-2282DA066 | 1 |
|  |  |  | DS1115-2282DA067 | 1 |
|  |  |  | DS1115-2282DA068 | 1 |
|  |  |  | DS1115-2282DA069 | 1 |
|  |  |  | DS1115-2282DA070 | 1 |
|  |  |  | DS1115-2282DA071 | 1 |
|  |  |  | DS1115-2282DA072 | 1 |
|  |  |  | DS1115-2282DA073 | 1 |
|  |  |  | DS1115-2282DF060 | 1 |
|  |  |  | DS1115-2282DF061 | 1 |
|  |  |  | DS1115-2282DF062 | 1 |
|  |  |  | DS1115-2282DF063 | 1 |
|  |  |  | DS1115-2282DF064 | 1 |
|  |  |  | DS1115-2282DF065 | 1 |
|  |  |  | DS1115-2282DF066 | 1 |
|  |  |  | DS1115-2282DF067 | 1 |
|  | Landsat 5 | 31/08/1998 | LT51470371998243XXX01 | 1 |
|  |  | 31/08/1998 | LT51470381998243XXX01 | 1 |
|  | Landsat 8 | 30/08/2015 | LC81470372015242LGN00 | 1 |
|  |  | 15/09/2015 | LC81470382015258LGN00 | 1 |
|  | Hexagon KH9 | 24/11/1973 | DZB1207-500047L023001 | 1,2 |
|  | Hexagon KH9 |  | DZB1207-500047L024001 | 1,2 |
| Central East | Hexagon KH9 | 20/12/1975 | DZB1211-500057L036001 | 1,2 |
|  | Hexagon KH9 |  | DZB1211-500057L037001 | 1,2 |
|  | Landsat 7 |  | LE71390412000361SGS00 | 1 |
|  | Sentinel-2A | 17/10/2017 | L1C_T45RXM_A003205_20171017T044824 | 1 |
|  |  | 17/10/2017 | L1C_T45RXL_A003205_20171017T044824 | 1 |
| Central West 1 | Hexagon KH9 | 12/12/1975 | DZB1211-500032L004001 | 1,2 |
|  | Hexagon KH9 |  | DZB1211-500032L005001 | 1,2 |
|  | Landsat 5 | 04/09/1998 | LT51430401998247BIK00 | 1 |
|  |  | 04/09/1998 | LT51430391998247BIK00 | 1 |
|  | Sentinel-2A | 11/09/2017 | L1C_T44RPU_A011599_20170911T051722 | 1 |
|  |  | 11/09/2017 | L1C_T44RPT_A011599_20170911T051722 | 1 |
| Central West 2 | Hexagon KH9 | 04/11/1974 | DZB1209-500033L022001 | 1,2 |
|  | Hexagon KH9 |  | DZB1209-500033L023001 | 1,2 |
|  | Landsat 7 | 15/12/2000 | LE71420402000350SGS00 | 1 |
|  | Sentinel-2A | 08/10/2017 | L1C_T45RTN_A011985_20171008T050247 | 1 |
|  | Sentinel-2B | 18/09/2018 | L1C_T45RTM_A008010_20180918T050419 | 1 |

**Supplementary Table 4.** A comparison of geodetic mass balance studies covering the main arc of the Himalaya over a comparable period to our SRTM-HMA DEM mass balance dataset.

| **Himalayan region** | **Glacier area (km^2^)/Number** | **Datasets** | **Mass balance** | **Study period** | **Study** | **Notes** |
| --- | --- | --- | --- | --- | --- | --- |
| West | 2527/356 | SRTM Vs HMA DEMs (WV & Geoeye) | **-0.40 ± 0.06** | 2000-2016 | This work |  |
|  | - | IceSat | -0.32 ± 0.06 | 2003-2008 | Kääb et al. (2012) |  |
|  | 2110/- | SRTM Vs SPOT 5 | -0.45 ± 0.14 | 1999-2011 | Gardelle et al. (2013) |  |
|  | 1796/- | SRTM Vs TanDEM-X | -0.52 ± 0.32 | 2000-2012 | Vijay and Braun (2016) | Estimate for Lahaul Spiti |
|  | 4477/705 | ASTER | -0.37 ± 0.09 | 2000-2016 | Brun et al. (2018) |  |
|  | 1569/1164 | SRTM Vs TanDEM-X | -0.43 ± 0.24 | 2000-2012 | Vijay and Braun (2018) | Estimate for Jammu and Kashmir West |
|  | - | ASTER | -0.45 ± 0.11 | 2000-2016 | Maurer et al. (2019) |  |
|  | - | SRTM-ASTER | -0.30 ± 0.10 | 2000-2013 | Mukherjee et al. (2018) | Estimate for Lahaul Spiti |
| Central West 1 | 682/138 | SRTM Vs HMA DEMs (WV & Geoeye) | **-0.41 ± 0.10** | 2000-2016 | This work |  |
|  | 910/- | SRTM Vs SPOT 5 | -0.32 ± 0.13 | 1999-2011 | Gardelle et al. (2013) |  |
|  | - | IceSat | -0.37 ± 0.15 | 2003-2009 | Kääb et al. (2015) |  |
|  | - | ASTER | -0.34 ± 0.09 | 2000-2016 | Brun et al. (2018) | Value taken from their broader ‘West Nepal’ region |
|  | - | ASTER | -0.38 ± 0.15 | 2000-2016 | Maurer et al. (2019) |  |
| Central West 2 | 871/191 | SRTM Vs HMA DEMs (WV & Geoeye) | **-0.26 ± 0.11** | 2000-2016 | This work |  |
|  | 470/41 | SRTM Vs SETSM (WV) | -0.21 ± 0.16 | 2000-2013 | Robson et al. (2018) |  |
|  | - | ASTER | -0.34 ± 0.20 | 2000-2016 | Brun et al. (2018) | Value taken from their ‘East Nepal’ region |
| Central 1 | 829/138 | SRTM Vs HMA DEMs (WV & Geoeye) | **-0.37 ± 0.11** | 2000-2016 | This work |  |
|  | 117/7 | Multiple optical imagery sources | -0.38 ± 0.17 | 2006-2015 | Ragettli et al. (2016) |  |
|  | - | ASTER | -0.34 ± 0.20 | 2000-2016 | Brun et al. (2018) | Value taken from their ‘East Nepal’ region |
| Central 2 | 979/125 | SRTM Vs HMA DEMs (WV & Geoeye) | **-0.37 ± 0.12** | 2000-2016 | This work |  |
|  | 50/10 | ASTER Vs Cartosat-1 | -0.79 ± 0.52 | 2002-2007 | Bolch et al. (2011) |  |
|  | 183/97 | Map-DEM Vs ASTER DEM | -0.40 ± 0.25 | 1992-2008 | Nuimura et al. (2012) |  |
|  | - | IceSat | -0.31 ± 0.14 | 2003-2008 | Kääb et al. (2015) |  |
|  | 1460/- | SRTM Vs SPOT 5 | -0.26 ± 0.14 | 1999-2011 | Gardelle et al. (2013) |  |
|  | 706/32 | SRTM Vs SETSM (WV) | -0.52 ± 0.22 | 2000-2016 | King et al. (2017) |  |
|  | - | ASTER | -0.33 ± 0.20 | 2000-2016 | Brun et al. (2018) | Value taken from their ‘East Nepal’ region |
|  | 700/30 | SRTM Vs TerraSAR-X/TanDEM-X | -0.38 ± 0.04 | 2000-2012 | Li et al (2018) |  |
|  | - | ASTER | -0.48 ± 0.03 | 2000-2016 | Maurer et al. (2019) |  |
| Central East | 599/128 | SRTM Vs HMA DEMs (WV & Geoeye) | **-0.37 ± 0.11** | 2000-2016 | This work |  |
|  | - |  | **-0.41 ± 0.11** | 2000-2016 | Maurer et al. (2019) |  |
| East | 746/148 | SRTM Vs HMA DEMs (WV & Geoeye) | **-0.43 ± 0.12** | 2000-2016 | This work |  |
|  | - | IceSat | -0.52 ± 0.16 | 2003-2008 | Kaab et al. (2012) |  |
|  | 1384/- | SRTM Vs SPOT 5 | -0.22 ± 0.13 | 1999-2011 | Gardelle et al. (2013) |  |
|  | 1239/187 | ASTER | -0.43 ± 0.20 | 2000-2016 | Brun et al. (2018) |  |
|  | - | ASTER | -0.51 ± 0.15 | 2000-2016 | Maurer et al. (2019) |  |
| Far East | 216/51 | SRTM Vs HMA DEMs (WV & Geoeye) | **-0.54 ± 0.20** | 2000-2016 | This work |  |
|  |  | IceSat | -0.76 ± 0.20 | 2003-2008 | Kaab et al (2015) | Value taken from their broader ‘Bhutan’ region |
|  | 2291/- | ASTER | -0.42 ± 0.20 | 2000-2016 | Brun et al. (2018) | Value taken from their broader ‘Bhutan’ region |

**Supplementary Table 5**. Comparison of mass balance estimates for glaciers depending on their debris cover and terminus type for our contemporary mass balance dataset (2000-‘2015).

| **Terminus type** | **Mass balance of clean-ice glaciers** | **Mass balance of debris-covered glaciers** |
| --- | --- | --- |
| Lake-terminating | -0.51±0.12 (n=64) | -0.67±0.15 (n=43) |
| Land-terminating | -0.35 ±0.12 (n=1008) | -0.41 ±0.12 (n=160) |

**Supplementary Table 6.** Comparison of Glacier terminus retreat rates (all in m a^-1^) across different regions and for different periods. The uncertainty associated with retreat rates from ‘~1974-2000 is ± 1.1 m a^-1^, for 2000-2017 it is ± 1.4 m a^-1^, and for ~1974-2018 it is ± 0.6 m a^-1^.

|  | **Lake-term.** | **Land-term.** | **All** | **Lake-term.** | **Land-term.** | **All** | **All** |
| --- | --- | --- | --- | --- | --- | --- | --- |
| **Himalayan Region** | **~1974-2000** | **~1974-2000** | **~1974-2000** | **2000-2018** | **2000-2018** | **2000-2018** | **~1974-2018** |
| West | 15.39 | 9.03 | 9.48 | 33.71 | 10.14 | 11.83 | 10.60 |
| Central West 1 | 15.17 | 6.87 | 9.89 | 30.38 | 7.62 | 16.49 | 12.42 |
| Central West 2 | 13.02 | 7.65 | 8.67 | 14.57 | 11.61 | 12.17 | 10.13 |
| Central 1 | 19.35 | 6.53 | 8.91 | 35.65 | 11.40 | 15.89 | 11.82 |
| Central East | 16.67 | 6.46 | 8.45 | 24.72 | 9.98 | 12.86 | 10.22 |
| East | 15.90 | 6.17 | 8.78 | 22.21 | 11.73 | 14.53 | 10.97 |
| Mean | 15.92 | 7.12 | 9.03 | 26.87 | 10.41 | 13.96 | 11.03 |

**Statistical analyses of mass balance datasets**

Using unpaired, two-tailed t-tests, the results of which are shown below and in Supplementary Table 7, we have examined the differences in mass balance estimates for our full samples of lake and land-terminating glaciers, as well as the samples of mass balance data for debris-covered and clean ice glaciers, over both time periods.

We carried out the same tests at the scale of our sub regions but could not apply the tests to data for all sub regions because of the limited number of lake-terminating or debris-covered glaciers, especially in our dataset covering the period ~1974-2000 (Supplementary Table 8).

**Supplementary Table 7**. The results of unpaired, two tailed t-tests to examine the characteristics of differences in mass balance data for glaciers of different terminus type and debris cover extent over both time periods of our study.

| **Test case** | **P-value** | **Mean diff.** | **95% confidence range** | **t-value** |
| --- | --- | --- | --- | --- |
| Lake Vs land ~1974-2000 | 0.0001 | -0.09 | -0.17 to -0.06 | 4.07 |
| Lake Vs land 2000-~2015 | 0.0001 | -0.18 | -0.24 to -0.16 | 10.04 |
| Debris Vs clean ~1974-2000 | 0.0037 | -0.07 | -0.13 to -0.02 | 2.91 |
| Debris Vs clean 2000-~2015 | 0.0017 | -0.06 | -0.10 to -0.02 | 3.14 |

Considering the results of our statistical analyses of our full samples of mass balance data, we can make the following statements about the differences between mass balance estimates for lake and land-terminating glaciers, and glaciers with or without substantial debris cover.

The low p-values associated with all test cases suggests that the observed differences are highly unlikely to have occurred due to random sampling. In all cases, p<0.05, although these cannot be used alone to state that a statistically significant difference exists between the different samples of mass balance data we have tested. Small p-values are commonly derived through the comparison of differences in particularly large datasets and small differences of no real interest, for example those between debris-covered and clean-ice glacier mass balance, appear statistically significant (Gardner and Altman, 1986). We do note that the p-values associated with the differences between lake and land-terminating cases are an order of magnitude smaller than for the debris versus clean-ice tests.

The mean differences in the mass balance of lake- versus land-terminating glaciers are substantial when compared to the regional mean mass loss rates (Table 1 in the main manuscript) of glaciers across the region. The mean difference in mass loss between glaciers of different terminus types has doubled over our full study period. The mean difference in the mass loss rate of debris-covered and clean-ice glaciers did not increase over our study period and so we can state that the presence or absence of debris cover had no significant impact on mass loss rates over our full study period.

Large t-values occur when sample data is extremely dissimilar from the null hypothesis (that there is no difference in the mass balance of lake and land-terminating glaciers, for example). The largest t-values are associated with terminus type dependant tests; thus, we can say that there is most evidence for the influence of terminus type on mass balance estimates, especially so for the period 2000-~2015. There is little difference in the t-values for the debris cover versus clean test cases, thus there is less evidence for the influence of debris cover on mass balance estimates.

**Supplementary Table 8**. The results of unpaired, two tailed t-tests to examine the characteristics of differences in mass balance data for glaciers of different terminus type and debris cover extent over both time periods of our study. Blocked out grey cells represent sub-regions where insufficient (<15) mass balance difference estimates were available for lake-terminating or debris-covered glaciers to allow for the use of a t-test.

| **Test** | **p-value** | **Mean diff (m w.e.a^-1^)** | **95% confidence interval** | **t-value** |
| --- | --- | --- | --- | --- |
| Lake Vs land 2000-2015 West |  |  |  |  |
| Lake Vs land 2000-2015 Central West 1 | 0.0001 | -0.29 ± 0.07 | -0.42 to -0.15 | 4.20 |
| Lake Vs land 2000-2015 Central West 2 |  |  |  |  |
| Lake Vs land 2000-2015 Central 1 | 0.0087 | -0.14 ± 0.05 | -0.25 to -0.04 | 2.65 |
| Lake Vs land 2000-2015 Central 2 | 0.0001 | -0.19 ± 0.03 | -0.26 to -0.13 | 5.88 |
| Lake Vs land 2000-2015 Central East | 0.0015 | -0.13 ± 0.04 | -0.21 to -0.05 | 3.21 |
| Lake Vs land 2000-2015 East | 0.0001 | -0.28 ± 0.05 | -0.38 to -0.18 | 5.42 |
| Lake Vs land 2000-2015 Far East |  |  |  |  |
| Lake Vs land 1974-2000 West |  |  |  |  |
| Lake Vs land 1974-2000 Central West 1 |  |  |  |  |
| Lake Vs land 1974-2000 Central West 2 |  |  |  |  |
| Lake Vs land 1974-2000 Central 1 | 0.0130 | -0.11 ± 0.04 | -0.20 to -0.02 | 2.51 |
| Lake Vs land 1974-2000 Central 2 |  |  |  |  |
| Lake Vs land 1974-2000 Central East | 0.0900 | -0.09 ± 0.05 | -0.19 to 0.01 | 1.70 |
| Lake Vs land 1974-2000 East | 0.0416 | -0.13 ± 0.04 | -0.26 to -0.01 | 2.05 |
| Lake Vs land 1974-2000 Far East |  |  |  |  |
| Debris Vs clean 2000-2015 West | 0.6349 | -0.01 ± 0.03 | -0.07 to 0.04 | 0.47 |
| Debris Vs clean 2000-2015 Central West 1 | 0.1772 | -0.11 ± 0.08 | -0.28 to 0.05 | 1.35 |
| Debris Vs clean 2000-2015 Central West 2 | 0.9594 | -0.00 ± 0.05 | -0.09 to 0.08 | 0.05 |
| Debris Vs clean 2000-2015 Central 1 | 0.0154 | -0.09 ± 0.04 | -0.17 to -0.01 | 2.44 |
| Debris Vs clean 2000-2015 Central 2 | 0.0001 | -0.08 ± 0.02 | -0.12 to -0.04 | 4.00 |
| Debris Vs clean 2000-2015 Central East | 0.1801 | -0.07 ± 0.05 | -0.18 to 0.03 | 1.34 |
| Debris Vs clean 2000-2015 East | 0.0058 | -0.17 ± 0.05 | -0.29 to -0.05 | 2.79 |
| Debris Vs clean 2000-2015 Far East | 0.3758 | 0.11 ± 0.12 | -0.13 to 0.35 | 0.89 |
| Debris Vs clean 1974-2000 West | 0.0676 | -0.15 ± 0.08 | -0.31 to 0.01 | 1.91 |
| Debris Vs clean 1974-2000 Central West 1 |  |  |  |  |
| Debris Vs clean 1974-2000 Central West 2 | 0.2675 | -0.04 ± 0.04 | -0.03 to 0.12 | 1.11 |
| Debris Vs clean 1974-2000 Central 1 | 0.2826 | -0.03 ± 0.03 | -0.10 to 0.03 | 1.07 |
| Debris Vs clean 1974-2000 Central 2 |  |  |  |  |
| Debris Vs clean 1974-2000 Central East |  |  |  |  |
| Debris Vs clean 1974-2000 East | 0.1286 | -0.09 ± 0.06 | -0.21 to 0.02 | 1.52 |
| Debris Vs clean 1974-2000 Far East |  |  |  |  |

Considering the results of our statistical analyses of our mass balance estimates for sub-regions, we can make the following statements about the differences between mass balance estimates for glaciers of different terminus type or debris cover.

Again, p<0.05 for all test cases where we assessed mass balance differences depending on terminus type over the period 2000-~2015. We can state that a statistically significant difference exists in the mass balance of glaciers of different terminus type over this period for all sub-regions we tested. P>0.05 for two of the three regions where we could test mass balance differences depending on terminus type over the period ~1974-2000, suggesting that terminus type may not have had a statistically significant impact on ice loss rates over this time period.

In 5/8 cases, p>0.05 when we compared differences in mass balance depending on debris cover over the period 2000-~2015 (Table 2). In these sub-regions, we can state that debris cover did not have a statistically significant impact on glacier mass loss rates. In the other three cases, p-values ranged from 0.0001-0.0154 and mean differences ranged from -0.08 to -0.17, thus the impact of debris cover on mass loss is unclear.

The mean differences in the mass balance of lake versus land-terminating glaciers were again substantial over the period 2000-~2015. The mean differences of mass balance estimates of lake versus land terminating glaciers over this later time period were all above the terminus type dependant mean differences over the period ~1974-2000. The mean differences between debris-covered and clean-ice glaciers ranged from 0 to -0.17, but were generally small (Table 2).

The t-values produced by our tests of terminus type dependant mass balance differences at the sub-regional level were all high over the 2000-~2015 period (ranging from 2.65-5.42, Table 2). T-values were <2.51 in our three tests of terminus type dependant mass balance differences over the period ~1974-2000. The t-values associated with tests of debris cover dependant mass balance differences were <1.35 in 5/8 cases (Table 2). Our interpretation of these variable t-values mirrors our interpretation of their associated p-values.

Overall, the results of the t-tests support our conclusions that glacier mass balance is contrasting depending on terminus type, especially for our later study period (2000-~2015). The results of statistical analyses also suggest that there is little contrast in the mass balance of glaciers with and without substantial debris cover.


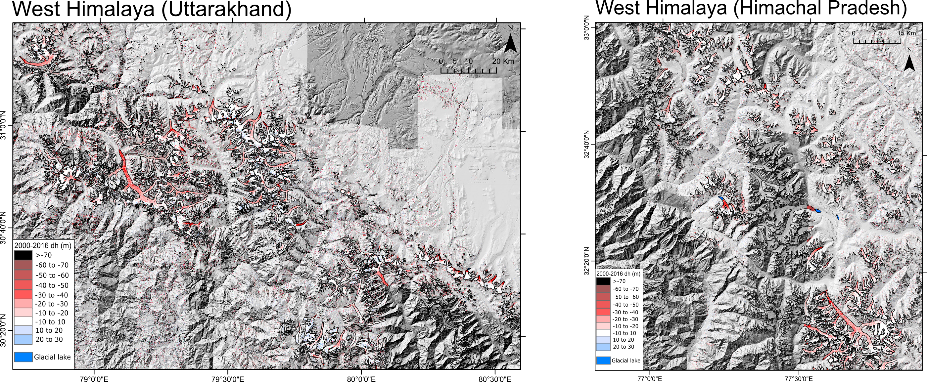


**Figure S2.** Surface elevation change over the West Himalaya from 2000-~2015. This figure was generated using ArcGIS, vers. 10.3 (http://www.esri.com/software/arcgis/arcgis-for-desktop). Used data sources: SRTM DEM (https://earthexplorer.usgs.gov/), High Mountain Asia DEM (Shean, 2017), Randolph Glacier Inventory, vers. 6.0 (RGI Consortium, 2017, www.glims.org/RGI), adjusted, Glacial lake inventory by Zhang et al. (2015), adjusted.


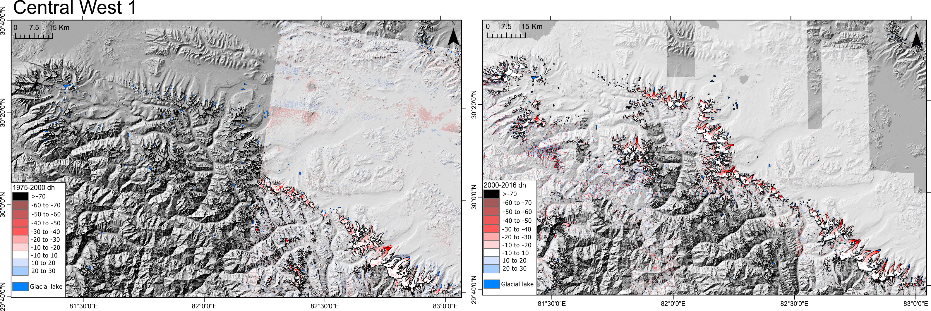


**Figure S3.** Surface elevation change over the Central West 1 region over the period ~1974-2000 (left) and 2000-~2015 (right). This figure was generated using ArcGIS, vers. 10.3 (http://www.esri.com/software/arcgis/arcgis-for-desktop). Used data sources: Hexagon KH-9 data and SRTM DEM (https://earthexplorer.usgs.gov/), High Mountain Asia DEM (Shean, 2017), Randolph Glacier Inventory, vers. 6.0 (RGI Consortium, 2017, www.glims.org/RGI), adjusted, Glacial lake inventory by Zhang et al. (2015), adjusted.


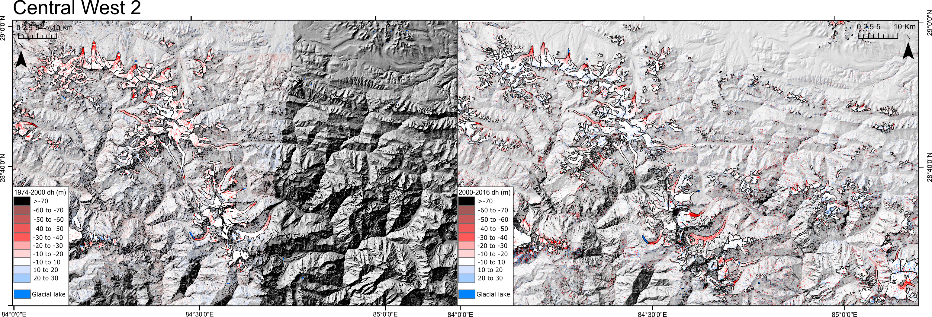


**Figure S4.** Surface elevation change over the Central West 2 region over the period ~1974-2000 (left) and 2000-~2015 (right). This figure was generated using ArcGIS, vers. 10.3 (http://www.esri.com/software/arcgis/arcgis-for-desktop). Used data sources: Hexagon KH-9 data and SRTM DEM (https://earthexplorer.usgs.gov/), High Mountain Asia DEM (Shean, 2017), Randolph Glacier Inventory, vers. 6.0 (RGI Consortium, 2017, www.glims.org/RGI), adjusted, Glacial lake inventory by Zhang et al. (2015), adjusted.


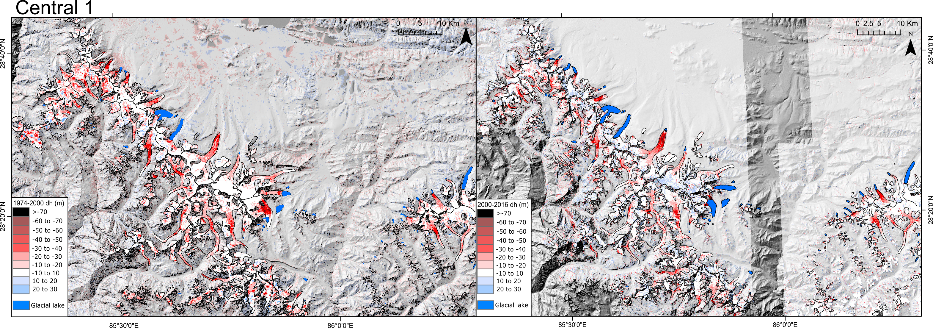


**Figure S5.** Surface elevation change over the Central 1 region over the period ~1974-2000 (left) and 2000-~2015 (right). This figure was generated using ArcGIS, vers. 10.3 (http://www.esri.com/software/arcgis/arcgis-for-desktop). Used data sources: Hexagon KH-9 data and SRTM DEM (https://earthexplorer.usgs.gov/), High Mountain Asia DEM (Shean, 2017), Randolph Glacier Inventory, vers. 6.0 (RGI Consortium, 2017, www.glims.org/RGI), adjusted, Glacial lake inventory by Zhang et al. (2015), adjusted.


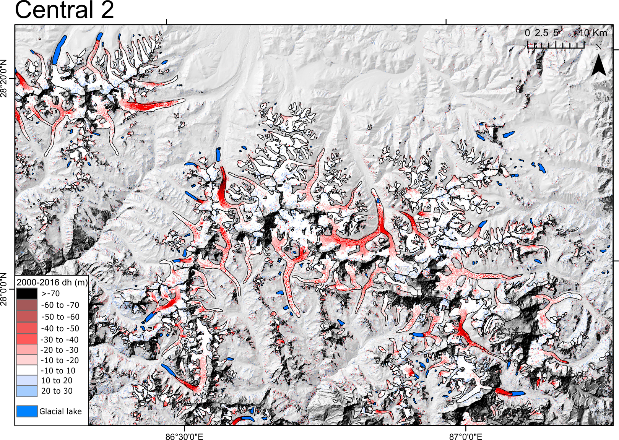


**Figure S6.** Surface elevation change over the Central 2 region over the period 2000-~2015. This figure was generated using ArcGIS, vers. 10.3 (http://www.esri.com/software/arcgis/arcgis-for-desktop). Used data sources: SRTM DEM (https://earthexplorer.usgs.gov/), High Mountain Asia DEM (Shean, 2017), Randolph Glacier Inventory, vers. 6.0 (RGI Consortium, 2017, www.glims.org/RGI), adjusted, Glacial lake inventory by Zhang et al. (2015), adjusted.


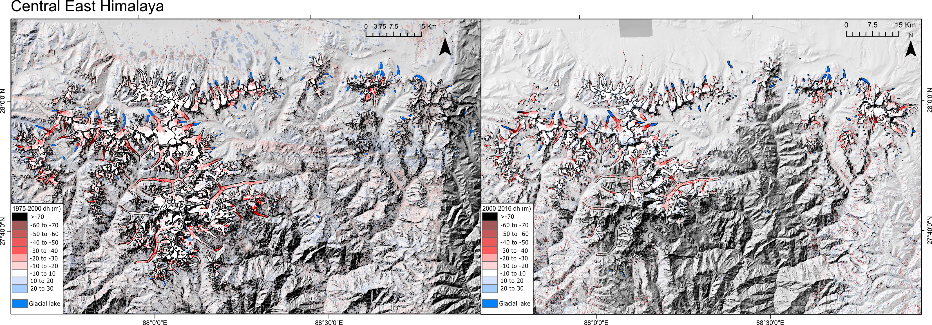


**Figure S7.** Surface elevation change over the Central East Himalaya over the period ~1974-2000 (left) and 2000-~2015 (right). This figure was generated using ArcGIS, vers. 10.3 (http://www.esri.com/software/arcgis/arcgis-for-desktop). Used data sources: Hexagon KH-9 data and SRTM DEM (https://earthexplorer.usgs.gov/), High Mountain Asia DEM (Shean, 2017), Randolph Glacier Inventory, vers. 6.0 (RGI Consortium, 2017, www.glims.org/RGI), adjusted, Glacial lake inventory by Zhang et al. (2015), adjusted.


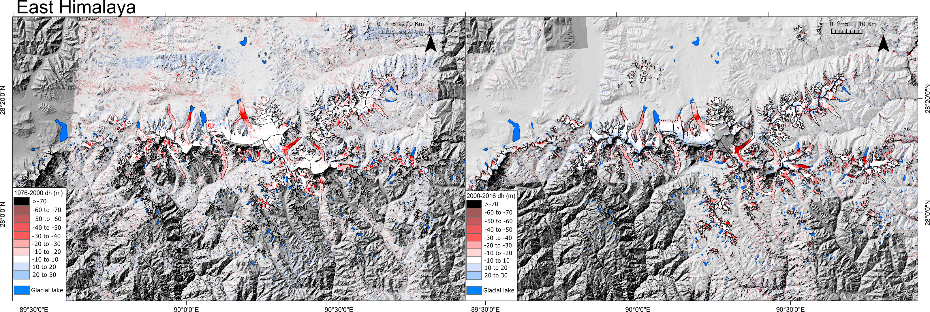


**Figure S8.** Surface elevation change over the East Himalaya over the period ~1974-2000 (left) and 2000-~2015 (right). This figure was generated using ArcGIS, vers. 10.3 (http://www.esri.com/software/arcgis/arcgis-for-desktop). Used data sources: Hexagon KH-9 data and SRTM DEM (https://earthexplorer.usgs.gov/), High Mountain Asia DEM (Shean, 2017), Randolph Glacier Inventory, vers. 6.0 (RGI Consortium, 2017, www.glims.org/RGI), adjusted, Glacial lake inventory by Zhang et al. (2015), adjusted.


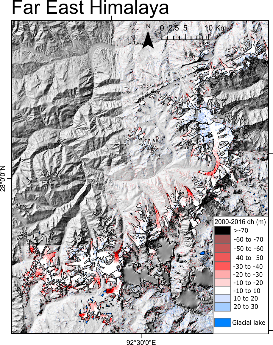


**Figure S9.** Surface elevation change over the Far East Himalaya over the period 2000-~2015. This figure was generated using ArcGIS, vers. 10.3 (http://www.esri.com/software/arcgis/arcgis-for-desktop). Used data sources: SRTM DEM (https://earthexplorer.usgs.gov/), High Mountain Asia DEM (Shean, 2017), Randolph Glacier Inventory, vers. 6.0 (RGI Consortium, 2017, www.glims.org/RGI), adjusted, Glacial lake inventory by Zhang et al. (2015), adjusted.

**References**

Bolch, T., Pieczonka, T. & Benn, D.I. Multi-decadal mass loss of glaciers in the Everest area (Nepal Himalaya) derived from stereo imagery. *Cryosphere* **5** 349-358 (2011).

Brun, F., Wagnon, P., Berthier, E., Shea, J., Immerzeel, W., Kraaijenbrink, P.D.A., Vincent, C., Reverchon, C., Shrestha, D. & Arnaud, Y. Ice-cliff contribution to the tongue-wide ablation of the Changri Nup Glacier, Nepal, central Himalaya. *Cryosphere*, **12**, 3439-3457 (2018).

Gardelle, J., Berthier, E., Arnaud, Y. & Kääb, A. Region-wide glacier mass balances over the Pamir-Karakoram-Himalaya during 1999-2011. *Cryosphere*. **7** 1263-1286 (2013).

Gardner, M.J. & Altman, D.G. Confidence intervals rather than P values: estimation rather than hypothesis testing. *Br. Med. J.* **292** 746-750 (1986).

Nuimura, T., Fujita, K., Yamaguchi, S. & Sharma, R.R. Elevation changes of glaciers revealed by multitemporal digital elevation models calibrated by GPS survey in the Khumbu region, Nepal Himalaya, 1992-2008. *J. Glacio*. **58** 648-656 (2012).

Kääb, A., Berthier, E., Nuth, C., Gardelle, J. & Arnaud, Y. Contrasting patterns of early twenty-first-century glacier mass change in the Himalayas. *Nature* **488** 495-498 (2012).

Kääb, A., Triechler, D., Nuth, C. & Berthier, E. Contending estimates of 2003-2008 glacier mass balance over the Pamir-Karakoram-Himalaya. *Cryosphere*, **9** 557-564 (2015).

King, O., Quincey, D.J., Carrivick, J.L. & Rowan, A.V. Spatial variability in mass loss of glaciers in the Everest region, central Himalayas, between 2000 and 2015. *Cryosphere*. **11** 407-426 (2017).

Li, G., Lin, H. & Ye, Q. Heterogeneous decadal glacier downwasting at the Mt. Everest (Qomolangma) from 2000 to ~2012 based on multi-baseline bistatic SAR interferometry. *Rem. Sens. Environ.* **206** 336-349 (2018).

Maurer, J., Schaefer, J.M., Rupper, S. & Corley, A. Acceleration of ice loss across the Himalayas over the past 40 years. *Sci. Adv*. **5** (2019).

McNabb, R., Nuth, C., Kӓӓb, A. & Girod, L. Sensitivity of glacier volume change estimation to DEM void interpolation. *Cryosphere* **13** 895-910 (2019).

Mukherjee, K., Bhattacharya, A., Pieczonka, T., Ghosh, S. & Bolch, T. Glacier mass budget and climatic reanalysis data indicate a climate shift around 2000 in Lahaul-Spiti, western Himalaya. *Climatic Change.* **148**(1–2), 219–233 (2018).

Ragettli, S., Bolch, T., & Pellicciotti, F. Heterogeneous glacier thinning patterns over the last 40 years in Langtang Himal, Nepal, *Cryosphere*. **10** 2075-2097 (2016).

RGI Consortium (2017). Randolph Glacier Inventory – A Dataset of Global Glacier Outlines: Version 6.0: Technical Report, Global Land Ice Measurements from Space, Colorado, USA. Digital Media.

Robson, B.A. Nuth, C., Nielsen, P.R., Girod, L., Hendrickx, M. & Dahl, S.O. Spatial variability in patterns of glacier change across the Manasalu range, Central Himalaya. *Front. Earth. Sci*. **6** (2018).

Schneider, A., Jost, A., Coulon, C., Silvestre, M., Théry, S. & Ducharne, A. Global-scale river network extraction based on high-resolution topography and constrained by lithology, climate, slope, and observed drainage density. Geophys. Res. Lett 44 (6), 2773–2781. (2017).

Shean, D. High Mountain Asia 8-meter DEM Mosaics Derived from Optical Imagery, Version 1. Boulder, Colorado USA. NASA National Snow and Ice Data Center Distributed Active Archive Center. (2017).

Surazakov, A.B., & Aizen, V.B. Positional accuracy evaluation of declassified Hexagon KH-9 mapping camera imagery. Photogrammetric Engineering and Remote Sensing, 76, 603–608 (2010).

Vijay, S. & Braun, M. Elevation change rates of glaciers in the Lahaul-Spiti (Western Himalaya, India) during 2000-2012 and 2012-2013. *Rem. Sens.* **8** (2016).

Vijay, S. & Braun, M. Early 21^st^ century spatially detailed elevation changes of Jammu and Kashmir glaciers (Karakoram-Himalaya). *Glob. Plan. Change.* **165** 137-146 (2018).

Zhang, G., Yao, T., Xie, H., Wang, W. & Yang, W. An inventory of glacial lakes in the Third Pole region and their changes in response to global warming. *Global Planet. Change* **131** 148–157 (2015).
